# Ayurveda and Yoga management of chronic alcoholism sequelae - A case report

**DOI:** 10.1016/j.jaim.2023.100862

**Published:** 2024-01-18

**Authors:** Aqsa Zarin Khan, Jibi Varghese, Shweta Kodre, Mohini Niware, Snehal Pansare, Shreya Bhatia, Samkit Shah, Manna Mathew

**Affiliations:** a Department of Kayachikitsa, Dr D.Y. Patil College of Ayurved & Research Centre, Pimpri, Pune, India; b Ayurveda practitioner at Ayur Arogyam, Nigdi, Pune, India

**Keywords:** *Rasayana*, *Vikshay*, Chronic alcoholism, Thiamine deficiency, Ayurveda, Yoga

## Abstract

Alcohol has always been a component in the dietary pattern of human civilization. It is widely used in society for celebration and socialization. Alcohol abuse is among the most serious problems in public health characterized by uncontrolled drinking which causes physical and emotional dependence on alcohol. Chronic alcoholics are at a higher risk of developing vitamin B1 deficiency due to malabsorption, poor diet, and an increased demand for nutrition. Vitamin B1(Thiamine) is an essential nutrient required for the body's energy metabolism and proper functioning of the nervous system. A person who excessively consumes *madya* (alcohol) and then abruptly discontinues drinking and takes recourse to drinking excess *madya* once again, suffers from *Madatyaya Upadrava(*chronic alcoholism) that is *Vikshay*. Here is a case report of an alcoholic patient who ceased drinking and then resumed alcohol in large amounts. He presented with symptoms of generalized weakness, body ache, aphasia, confusion, fever (on and off), thirst, cough, headache, and numbness. The patient underwent a two-month treatment regimen that combined *Satvavajay Chikitsa*, Yoga, and *Shaman Chikitsa* involving *Rasayana* medications and procedures including *snehan* (Oleation), *swedan* (fomentation), *nabhi puran* (filling oil with navel), *nasya* (nasal administration), *shirodhara* (continuous flow of liquid on head) and *basti* (medicated enema). The intervention outcome showed relief from the aforementioned symptoms and improvement in both symptoms and GCS(Glasgow coma scale) score. This treatment approach aimed to promote vitality, longevity, and an overall sense of balance and well-being. There are not many corroborating cases being reported and managed with Ayurveda. This case report highlights transforming health through the cumulative effects of *Rasayana* medicines, *panchakarma,* and yoga.

## 1 Introduction

Liquor is described as *amritam* (elixir) when consumed adequately but when consumed improperly causes disorders depicting its intoxication symptoms [1]. Similarly, a poison, when used sensibly, can be life-saving. Heart is the site for channeling the *Rasa dhatu* (the nutritional fluid produced immediately after digestion)*, Satva* (mental strength), *Buddhi* (intellect), *Indriya* (senses), *Aatma* (self) and *Ojas* (vital essence of the body). *Madya* with its ten qualities affects the *hridaya* by counteracting the ten qualities of *ojas*. The excess drinking of liquor and gradual impediment of *ojas* affect the heart and the *dhatu* located at the heart [2]. These properties of alcohol are *laghu* (lightness), *ruksa* (dry), *tikshna* (penetrating), *ushna* (hot), *suksma* (diffusing), *amla* (sour), *vyavaya* (absorbs without undergoing digestive changes), *asukari* (fast acting), *vikasi* (breaks the internal tissue barriers) and *visada* (clearing the channels). In this case report, the patient was economically poor and was a labourer who used to carry loads.*.* The patient consumed excessive amounts of alcohol without an adequate diet. This resulted in his malnourishment. Patient gradually started experiencing body ache, aphasia, confusions, fever,thirst, cough, headache, numbness. In Ayurveda, these symptoms state a condition that can be correlated with *Vikshay* (*Madatyaya Upadrava*). Also as stated by Charaka, the one who starts consumption of liquor in excess quantity after abstaining, suffers from the disease called *Vikshay* [3]. The treatment of *Vikshay* has been indicated in Charaka *samhita* as per *vatika madatyaya*. Ayurvedic oral medication with *panchakarma* along with *yoga* and lifestyle modification has shown significant improvement in reversing the pathological condition.

**Table 1:** Showing the *Samprapti Ghatak* involved in the pathogenesis of *Madatyaya-Vikshay.*

Samprapti Ghatak	
*Dosha*	*Tridosha*
*Dushya*	*Rasa, Rakta, Meda, Majja,*
*Srotas*	*Rasa, Rakta, Medovaha, Majjavaha,* *Manovaha, Annovaha, Sanjnavaha*
*Sroto Dushti*	*Sanga*
*Agni*	*Mandagni*
*Udbhav Asthana*	*Amashaya*
*Sancarasthana*	*Sarva rasavahini, Sanjnyavahini*
*Adhisthana*	*Hridaya*
*Rogamarga*	*Madhyama*

**Table 2:** Laboratory tests on admission

Type	Value	Unit
Vitamin B1 (Thiamine)	1	μg/dL
AST (aspartate aminotransferase)	35.6	U/L
ALT (alanine aminotransferase)	32.1	U/L
Bilirubin, total	1.2	mg/dL
Bilirubin, conjugated (direct)	0.41	mg/dL
Bilirubin Indirect	0.79	mg/dL
ALP (alkaline phosphatase)	188	U/L
Protein, total	6.12	g/dL
Albumin	3.56	g/dL
Globulin	2.56	g/dL
A/G Ratio	1.39	
Blood Urea	44.12	mg/dL
Creatinine	1.38	mg/dL
Uric acid	3.8	mg/dL
Sr.Sodium	141.5	mEq/L
Sr.Potassium	4.05	mEq/L
Sr.Chloride	105.8	mEq/L
Cholesterol, total	141.4	mg/dL
Triglycerides	120.7	mg/dL
Cholesterol, HDL	41.3	mg/dL
Cholesterol, LDL	76	mg/dL
Cholesterol, VLDL	24.1	mg/dL
S.Cholesterol/HDL Ratio	3.4	
Hb(haemoglobin)	11.8	g/d
WBC	5700	cmm
Platelet Count	0.88	lac/cumm
FBS (fasting blood sugar)	91	mg/dL
PPBS (postprandial blood sugar)	129	mg/dL
Urine (Routine and Microscope)	Within normal limits	
ECG	Normal Sinus Rhythm	
X-Ray(Chest)	No any significant abnormality detected	
USG	Grade 1 Fatty Liver

**Table 3:** Yoga modules for holistic healing and alcohol addiction recovery

Type of Practice	Practices
Prayer	Starting Yoga with prayer
Loosening Practices	Fingers (Clench fist and open), Wrist (bending and rotation), Elbow movements, Shoulder rotation, Neck movements (Up-down, Right and Left movements), Hip loosening exercise, Knee flexion, Ankle movement, Toes movement
Breathing Practices (*Pranayama*)	*Anulom Vilom Pranayama*, *Ujjayi Pranayama*, *Bhramari Pranayama*
Simple Yogic Poses (*Asana*)	*Shavasana*, *Tadasana*, *Katichakrasana*, *Balasana*, *Shashankasana*, *Virasana* (Thinker's pose), *Gomukhasana*, *Sinhasana*, *Makarasana*.
Meditation	Om chanting

## 2 Patient information

A 71-year-old male patient reported to the outpatient department of *Kayachikitsa* (general medicine ayurveda) of Dr D.Y. Patil College of Ayurved and Research Centre, Pimpri-Pune. He presented with generalized weakness, body ache, aphasia, confusion, fever (on and off), thirst, cough, headache and numbness. Family members claimed that the patient used to speak about fictitious incidents which never occurred like, he was a player for the IPL matches, or sometimes claiming himself as a film actor etc. The history of the Patient revealed that he was a chronic alcoholic and used to consume 180 ml of country liquor from the age of 14 years. He was habitual to drink alcohol frequently and before two years in the year 2020 he abstained from drinking for some months, and later again started re-consuming liquor daily. The patient had no history of any systemic illness like hypertension, diabetes mellitus, tuberculosis, etc. He was not on any medication. He had taken some ayurvedic medication in his village, as he fell ill with excessive alcohol consumption and later discontinued. No data was available about his medication. On examination the patient's pulse was 80/minute, blood pressure 130/80 mm of hg and weight 55 kg, with height 174 cm. Table 2 represent patient's laboratory investigations on admission.

## 3 Clinical findings

### 3.1 *Asthasthana pariksha/*eightfold examination

*Nadi* (Pulse) - *Vata Pitta* dominant, *Mutra* (Urine) - Normal, *Mala* (Bowels) - Constipation, *Jivha* (Tongue) - Coated, *Shabda* (Speech) - Slurred, *Druka* (Eyes) - No any abnormality, *Aakruti* (Built) Lean built, *Sparsha* (Skin) - Dry.

### 3.2 Systemic examination

CNS - Confused, GCS Score - 13, CVS – S1S2 audible, RS - Air entry bilaterally equal, Cerebellar - No Gait ataxia, No Tandem gait, No Dysmetria, No Dysdiadochokinesia.

## 4 Diagnosis

### 4.1 Diagnostic criteria

A Chronic alcoholic patient who resumed consumption of liquor in excess quantity after abstaining from it. Also patient presents with signs and symptoms of *Madatyaya Upadrava Vikshay* is *angaruja* (severe pain in the body), *aspashtavaka* (throat disorders), *sammōha* (confusion), *jwara* (fever), *trishna* (thirst), *kasa* (cough), *shirashoola* (headache).

### 4.2 Etiopathogenesis

Excessive consumption of *madya* with inadequate diet and heavy work load all these causative factors that lead to vitiation of three *doshas* which lead to *annovoha srotodushti* and *agnimandya*. The *apachit* and *dushit ahar rasa* traverse and inflict the *Hridaya* causing *uttarotar dhatu shaithilya* and *ojo vikrutti.* Similarly, the *tikshna, ushna,* and *amla guna* of *madya* lead to *pitta* and *vata dushti* causing inflammation of the mucosal layer of the stomach and colon causing a reduction in the absorption of nutrients, especially vitamin B1(Thiamine), which causes symptoms like numbness in limbs, muscle weakness to mental and neurological deficits[4]. *Ruksha* and *Laghu guna* of *madya* vitiate the *vata dosha* leading to *Majja dhatu kshaya* causing symptoms of nutritional and neurological deficiency, body ache and weakness. It further inflicts the *Manovaha srotas* where *satva guna* is reduced and *raja tama guna* increases due to alcohol consumption leading to *Madatyaya* which further leads to *Vikshay* as *Upadrava.* Lifting heavy loads causes *Udan vayu dushti* leading to *vaka aspashtta* (aphasia), reduction of *Bala*(strength), *Oja, and* intelligence, along with a decrease in grasping power and memory [5]. Excessive alcohol consumption causes *Meda Dhatu Kshaya* which leads to *Balahani* (loss of strength). Table 1 shows the factors involved in the pathogenesis of *Vikshay.*

## 5 Therapeutic intervention

*Madatyaya Upadrava* or stages of *Dhwansaka* and *Vikshay* appears in *Ksheena* (emaciated) and *durbala* persons (weak). *Acharya* Charaka prescribed the treatment of *Vikshay* similar to *Vataja Madatyaya* [6]. The therapeutic approach involved *Satvavajay Chikitsa* and Yoga, *Shaman Chikitsa* and *Shodhan Chikitsa*.

### *5.1* *Satvavajay Chikitsa* and Yoga

*Acharya* Charaka quoted *Satvavajay Chikitsa* as ‘*Ahitebhyoarthebhyo Manonigraha*’ to control the mind and to withdraw the mind from unwholesome objects which can cause disease. *Satvavajay Chikitsa* can be used for the treatment of *Madatyaya* where the balance of *Raja* and *Tama guna* is achieved by the control of *dharneeya vega* (urges to be controlled). Yoga was used as *Satvavajay Chikitsa* which emphasises the principle of *‘Chitta Vritti Nirodha’* which means controlling the mind from different unwanted thoughts [7].

During the recovery period, alcoholics often experience cravings for alcohol due to the withdrawal of alcohol. Yoga emphasises mindfulness and increases body awareness with mental clarity and calmness. Table 3 shows the Yoga practices implemented for holistic healing and addiction recovery. This reduced the craving for alcohol with increased self-control. In this phase, *Ashtanga* yoga helps patients build strong willpower that helps them achieve sobriety by balancing the mind and body while executing various positions (*asana*). Regular practice of the *asanas* like *Shavasana*, *Tadasana*, etc. helped the patient to become more aware of his body and sensations, allowing him to develop a deeper understanding of his physical and emotional states. *Asana* helped the patient manage his emotions by reducing stress and promoting relaxation thus resisting the urge to drink alcohol. *Pranayama (*Breathing exercises) was also advised for calming the mind. It increases the circulation of blood to the brain and regulates breathing and movements, which improves self-regulation and impulse control [9]. Yoga intervention was started in the second week after the commencement of Ayurveda treatment. The yoga session lasted for 45 min. Practices implemented in the Yoga module are mentioned in the below table.

### 5.2 *Shamana Chikitsa*

The treatment plan for two months comprised the use of Capsule *Rasayana*, *Ashwagandha ghana vati*, *Yashtyadi Churna* (*Yashti, Pippali, Vacha, Aakarkarambh*), and *Shreekhandasava*. *Ekangaveer Rasa* was administered for one month, followed by a two-week pause, and then resumed for another two weeks.

### 5.3 *Shodhana Chikitsa*

The treatment regimen included *Snehan, Swedan, Nasya,* and *Nabhipuran* for two months, *Matra Basti* for three weeks with a one-week gap, and *Shirodhara* for one month. The patient received *Snehan* using lukewarm *Bala taila* for 20 min, followed by a 10-min *Swedan* using *Dashmoola Bharad* for two months, in the morning. The *Nasya* procedure was done by massaging *Bala taila* onto the face, neck, and forehead, followed by mild fomentation and then installation of *Bala taila* (6 *Bindu*) into both nostrils. The patient was advised to spit secretions if any. The procedure of *Shirodhara* was performed wherein, the patient was directed to lie down on a comfortable bed. *Jatamansi Taila* was then slowly and gently dripped onto his forehead and scalp in a rhythmic oscillating motion. The patient underwent *Matra basti* with 80 ml of *Bala taila* after breakfast, and later in the evening, *Nabhipuran* was performed by filling the navel with *Bala taila*.

## 6 Follow-up and outcomes

Severe body pain was evaluated using a VAS Scale. It was initially at 6 in the first three weeks, decreased to 3 in the following three weeks, and eventually reached 0 by the seventh week. The Mini-Mental State Examination (MMSE) was used to assess *Sammoha* (Confusions). In MMSE a score between 24 and 30 indicates no cognitive impairment, while a score between 18 and 23 suggests mild cognitive impairment and a score between 0 and 17 indicates severe impairment. In the initial six weeks, the score was between 18 and 23, indicating mild cognitive impairment. However, over the last seven and eight weeks, there was a gradual improvement in his mental status, and the score reached 28, indicating no cognitive impairment. *Shirashoola* (Headache) was evaluated using a VAS Scale. It registered a score of 3 in the first week and subsequently diminished to zero. In the first week, *Jwara* (fever) ranged from 100.5 to 102.2 °F with on-and-off onset. Subsequently, there were no more fever episodes. The vitamin B1 level rose from 1 μg/dL to 2 μg/dL during treatment. The PACS(Penn alcohol craving scale) is a unidimensional scale, assessing cravings for alcohol, including the frequency, intensity, and duration of cravings. It is a 5-item self-report questionnaire with scores for each response ranging from 0 to 6, and higher total scores suggest higher levels of alcohol craving. PACS scores of less than 15 indicate mild craving, 15–20 suggest moderately difficult cravings to resist, and more than 20 indicate very difficult cravings to resist. During the initial three weeks, the PACS score ranged from 15 to 20. Subsequently, in the following weeks (fourth, fifth, sixth, and seventh), the PACS score gradually decreased to 9, indicating the impact of Ayurvedic treatment. In the last week, the score reached 1, signifying the absence of craving.

Before treatment, the patient had *aspashtavaka* (aphasia) with reduced ability to speak or comprehend words which improved to normal speech after treatment in the last week. On admission, the patient exhibited intense thirst, which persisted despite consuming water during the initial two weeks. Subsequently, in the third and fourth weeks, the excessive thirst subsided after drinking water. Throughout the fifth, sixth, and seventh weeks, the thirst occurred at brief intervals, returning to normal intervals in the last week. During the initial first week, the patient had frequent coughing, which did slightly interfere with the usual daytime activities. In the second week, there was an occasional cough for short periods. However, starting from the third week, there were no episodes of coughing. During the initial five weeks, numbness was occasionally. However, from the sixth week onward, there was no occurrence of numbness. The symptoms mentioned above exhibited weekly improvement, culminating in overall relief by the eighth week.

## 7 Discussion

According to the assessment, the symptom of the patient was well managed by the holistic approach of Ayurveda and Yoga. The treatment principle of *Vikshay* is similar to *Vataja Madatyaya Chikitsa*. *Vata dosha* is pacified by internal Ayurvedic medicine along with Ayurvedic *therapies* and Yoga. Medicines like *Capsule Rasayan, Shreekhandasava,* and *Ekangaveer Rasa* pacified the vitiated *Vata dosha* improving the *agni* (digestive fire) and *bala* of the patient. *Yashtyadi Churna* enhanced his speech and memory and relieved his cough. *Panchakarma* was adopted on the principle of *Santarpana* and B*rimhana chikitsa*. *Snehan, Swedan* followed by *Nasya, Basti* and *Nabhipuran* enhanced the nourishment of the body and balanced the vitiated *doshas*. Yoga relieved his mental stress and brought up stability and mental well-being by improving the *Satva guna*. Proper counselling and the practice of Yoga improved his physical health by increasing strength, flexibility, and balance. This led to an increase in his confidence and self-efficacy, which improved his ability to resist alcohol cravings. After two months of treatment vitamin B1 increased from 1 μg/dL to 2 μg/dL and the GCS score improved from 13 to 15.

The rationale of treatment focused mainly on the principles of pacifying elevated *Vata dosha*, *Santarpana* and *Brimhana.* The probable mode of action of the treatment is as follows:

*Capsule Rasayana* has *Guduchi, Gokshura,* and *Amalaki. Guduchi* serves as a memory enhancer with lots of antioxidants promoting anti-ageing and immunity properties. It also supports liver health to regenerate liver tissue and helps to eliminate toxins from the body. It is beneficial in maintaining harmony for all three doshas, especially *Vata* and *Pitta*. It is found effective in treating neurological disorders like Amyotrophic lateral sclerosis, Parkinsonism, Dementia, Motor and Cognitive deficits and neuron loss in the spine and Hypothalamus. It is also used in the treatment of fever [8]. *Gokshura* is an antioxidant that reduces inflammation and pain and improves libido and weakness of the nervous system. Usually given in urinary problems but it also strengthens cardiac muscles and prevents atherosclerosis. Also effective in improving memory, concentration and alertness [9]. *Amalaki* is loaded with Vitamin C, a powerful antioxidant with anti-ageing properties and improving immunity. It eliminates stress and weakness maintaining healthy function of the liver, heart, brain and lungs [10]. *Ashwagandha ghana Vati* modulates stress and anxiety, improves strength and immunity, improves cognition, and memory functions, and is rejuvenating, anti-inflammatory and antiarthritic. It is used in Ayurveda as a *Rasayana* or adaptogen [11].

*Yashtyadi Churna* has *Pippali, Yahtimadhu, Vacha,* and *Aakarkarambh. Pippali* is a good antioxidant with immunomodulatory, hepatoprotective, anti-inflammatory and analgesic properties [12]. *Yahtimadhu is* anti-inflammatory, improves immunity, relieves stress and soothes the respiratory ducts. It also improves liver functions [13]. *Vacha* has cardioprotective and neuroprotective activity thereby enhancing learning and memory-enhancing activities. It also has antidepressant activity along with antioxidant, anti-inflammatory and immunomodulatory effects. It improves speech, enhances voice tone and quality and hence is useful for throat disorders [14]. *Aakarkarambh* has anti-inflammatory, analgesic and sialogogue activity [15]; it is a nervine tonic that improves cognitive functions [16].

*Shreekhandasava* is generally used for the treatment of cases having adverse effects of alcohol or to neutralise the effect of intoxicating drugs or the hangover effect. It also reduces the *ksharta* (alkalinity) of the *ahara rasa* produced due to excessive alcohol intake. Though ironically it also contains alcohol [17]. It also reduces excessive thirst. *Ekangveer rasa* is used in neurological disorders, relieves pain and stimulates the nervous system. It pacifies *vata kapha dosha* and nourishes the body with its *rasayana* qualities [18].

Procedures like *Sarvanga Snehan* nourish the body, make it strong and relieve *Angaruja* (body ache) by pacifying *vata*. *Sarvanga Swedan* improves blood circulation in the body, clears *srotavrodha* and balances *VataKapha dosha. Snehan* and *Swedan* provide psychosomatic well-being. *Nasya* is the installation of medicated oil into the nostrils. *Nasya* with *Bala Taila* has *snigdha* properties that nourish *Tarpak Kapha* and thus promote healthy brain and nerve function. *Basti* is said to have multidimensional effects when administered*. Matra Basti* with *Bala Taila* pacifies the vitiated *Vata dosha and* nourishes *Majja dhatu* and promotes normal functioning of nerves*. Matra Basti* promotes the functioning of the normal bacterial flora of the colon, thus regulating the body's synthesis of vitamin B1 [19]*.* Studies have shown that after taking *Basti,* there is a decrease in pyruvic acid, a component of keto acid. This decrease in pyruvic acid leads to an increase in vitamin B1 levels [20]. *Shirodhara* with *Jatamansi Taila* provides a feeling of relaxation and well-being. It relieves headaches, and mental exhaustion and calms the nervous system. *Nabhi* is the seat of *Udan Vayu* and *Udan Vayu* is responsible for *Bala, Varna, Oja* and memory*.* The vitiation of *Udan Vayu* caused by lifting burlaps is pacified by instilling *Bala Taila* in *Nabhi,* which nourishes *Udan Vayu* and thus improves *Bala, Oja,* speech and memory [21].

## 8 Conclusion

Alcoholism is the inability to control drinking alcohol causing physical and emotional dependence on alcohol. In the initial stages, the body can repair itself but chronic alcoholism has deleterious effects on normal life and biochemical parameters which can be life-threatening. Alcoholism leads to different health and social problems contributing to the global burden of the disease. Thus holistic management with Ayurveda and Yoga in managing the case of chronic alcoholism sequelae (*Madatyaya Upadrava-Vikshay*) has shown promising results. The patient is advised to continue the ayurvedic medicines and regular follow-ups. However, this case encourages more case studies and research in this area.

## 9 Patient perspective

The patient was satisfied and acknowledged the improvement he experienced after the treatment. He willingly adopted every treatment protocol, followed Yoga therapy and carried the same throughout his admission. He endorsed the significant improvements in his social life and quality of life.

## Source of funding

None.

## Author contributions

1. K. A. -Conceptualization, Software, Validation, Formal analysis, Investigation, Data curation, Writing–original draft, Writing–review and editing, Visualization, Supervision, Project administration.

2. V. J. - Conceptualization, Software, Validation, Formal analysis, Investigation, Data curation, Writing–original draft, Writing–review and editing, Visualization, Supervision, Project administration.

3. S. K. - Software, Investigation, Data curation, Writing–original draft, Writing–review and editing.

4. M. N. -Software, Investigation, Data curation, Writing–original draft, Writing–review and editing.

5. S. P. - Software, Investigation, Data curation, Writing–original draft, Writing–review and editing.

6. S. B. -Software, Investigation, Data curation, Writing–original draft, Writing–review and editing.

7. S. S. -Software, Investigation, Data curation, Writing–original draft, Writing–review and editing.

8. M.M -Software, Investigation, Data curation, Writing–original draft, Writing–review and editing.

## Source of funding

No any funding received.

## Declaration of competing interest

The authors declare that they have no known competing financial interests or personal relationships that could have appeared to influence the work reported in this paper.

## References

[1] Kushwaha H. (2018).

[2] Bulusu S. (2017).

[3] Kushwaha H. (2018).

[4] Sudarsana S. (2019).

[5] Tripathi D. (2019).

[6] Kushwaha H. (2018).

[7] Amin H., Sharma R. (2015). Nootropic efficacy of Satvavajaya Chikitsa and Ayurvedic drug therapy: a comparative clinical exposition. Int J Yoga.

[8] Saha S., Ghosh S. (2012). Tinospora cordifolia: one plant, many roles. Ancient Sci Life.

[9] Akram M., Asif M. (2011). A simple RP-HPLC method for determination of lornoxicam in human plasma. J Med Plants Res.

[10] Baliga M.S., Dsouza J.J. (2011). Amla (Emblica officinalis Gaertn), a wonder berry in the treatment and prevention of cancer. Eur J Cancer Prev.

[11] Gopukumar K., Thanawala S. (2021).

[12] Biswas P., Ghorai M. (2022). Piper longum L.: a comprehensive review on traditional uses, phytochemistry, pharmacology, and health-promoting activities. Phytother Res.

[13] Pastorino G., Cornara L. (2018). Liquorice (Glycyrrhiza glabra): a phytochemical and pharmacological review. Phytother Res.

[14] Sharma V., Sharma R. (2020). Role of Vacha (acorus calamus linn.) in neurological and metabolic disorders: evidence from ethnopharmacology, phytochemistry, pharmacology and clinical study. J Clin Med.

[15] Manouze H., Bouchatta O. (2017). Anti-inflammatory, antinociceptive, and antioxidant activities of methanol and aqueous extracts of anacyclus pyrethrum roots. Front Pharmacol.

[16] Sujith K., Darwin C.R. (2012). Memory-enhancing activity of Anacyclus pyrethrum in albino Wistar rats. Asian Pac. J. Trop. Dis..

[17] Sharma R., Jadhav M. (2021). Hepatoprotective activity of shrikhand asava-A PreclinicalStudy. Int.J.Pharm.Chem..

[18] Lohan V., Saini S. (2022). Management of ardita (Bell's Palsy) through Ayurveda Protocol-A Case Study. World J Pharmaceut Res.

[19] Dave A., Khagram R. (2010). Clinical effect of Matra Basti and Vatari Guggulu in the management of Amavata (rheumatoid arthritis). AYU.

[20] Vaidya Haridasa Shridhar Kasture (2010).

[21] Tripathi Dr (2019).

